# High-throughput sequencing of methylated cytosine enriched by modification-dependent restriction endonuclease MspJI

**DOI:** 10.1186/1471-2156-14-56

**Published:** 2013-06-18

**Authors:** Xiaojun Huang, Hanlin Lu, Jun-Wen Wang, Liqin Xu, Siyang Liu, Jihua Sun, Fei Gao

**Affiliations:** 1 Science & Technology Department, BGI-Shenzhen, Building No. 11, Bei Shan Industrial Zone, Yantian District, Shenzhen 518083, China; 2 College of Life Sciences, Wuhan University, Luojia Road No.16, Wuchang District, Wuhan 430072, China; 3 School of Life Science and Technology, University of Electronic Science and Technology of China, No.4, Section 2, North Jianshe Road, Chengdu 610054, China

**Keywords:** MspJI, DNA methylation, *Arabidopsis*

## Abstract

**Background:**

As a well-known epigenomic modification, DNA methylation is found to be common in plants and plays an important role in many biological processes. Relying on the unique feature of methylation-dependent digestion, the family of methylation-requiring restriction-like endonuclease, such as MspJI and its homologs, was suggested for a potential usage in methylation detection.

**Results:**

In this study, we combine MspJI digestion and electrophoretic band selection with next generation high-throughput sequencing technology to detect 5-methylcytosines in *Arabidopsis* genome. By developing a bioinformatics workflow to attribute the CNNR sites recognized by MspJI to the reference genome, we fulfilled the systematic assessment of this method.

**Conclusions:**

According to the assessment, here we provide the method for generating a detailed map of plant methylome that could be feasible, reliable and economical in methylation investigation.

## Background

DNA methylation occurs in all domains of life, from viruses to cellular organisms, serving for DNA protection in prokaryotes and regulation of gene expression in plants and animals [1-3]. DNA methylation in plants differs from that in mammals. In mammalian cells, methylation mainly occurs on the cytosines in CpG context, although non-CpG methylation is prevalent in embryonic stem cells as well [4]. While in plants, methylation occurs in both CpG and non-CpG contexts [5]. Besides, plant genomes are normally much larger than mammalian ones and contain a lot of repetitive sequences. Although the gold standard method whole genome bisulfite sequencing (WGBS) can be used to examine DNA methylation in single-base resolution, it requires large volume of sequencing data for confident DNA methylation calling. As a result, using WGBS for plant methylome profiling would be extremely costly. Thus, it is essential to develop a high-resolution and cost-effective methodology that can enrich the methylated sites, and accurately detect DNA methylation in plants.

In 2010, a unique family of methylation-requiring restriction-like (Mrr-like) endonucleases composed of MspJI and its homologs was reported in prokaryotes, and previous studies showed that their methylation-dependent digestion may protect host DNA against invading DNA [6]. Researchers announced that even if MspJI and its homologs are different in recognizing sites, similarities in cutting features are obvious such as the low frequency wobble by 1 base and a higher efficiency of enzyme digestion in the presence of oligonucleotide activators [6]. MspJI recognizes 5-methylcytosine (5 mC) in the context of ^m^CNNR (R = G or A) and introduces double-stranded breaks at fixed distances-N12∕N16 on the 3’ side of the mC, leaving a four-base 5’ overhang. Since mC lies in certain distance from the terminal of the digested fragments, the MspJI family of restriction enzymes digestion combined with deep sequencing can reveal credible methylation information in a genome, which has been demonstrated [7].

Theoretically, up to half of the total methylcytosines can be recognized by MspJI in a genome, which provides the possibility of interrogating enzyme-enrichment with high-throughput sequencing to decipher the representative DNA methylome in plants. In this study, we used MspJI as a representative of the enzyme family to recognize and enrich 5 mCs in the genome of model plant *Arabidopsis thaliana*. We size-selected the generated DNA fragments from MspJI digestion on agarose gel that may account for 25.03% of the total 5 mCs, including the majority of symmetrical ^m^CNNR sites and a portion of the asymmetrical ^m^CNNR sites, and used Illumina HiSeq2000 genome sequencer to sequence the enriched fragments. Especially, we developed corresponding bioinformatics tools for the analysis of mapping and recognition of 5 mCs in the enriched fragments. We compared our data with the methylome data generated by WGBS technology to assess this method, and further addressed the characteristics of *Arabidopsis* methylome which were in agreement with previous studies [8,9]. We thus comprehensively assessed the method for DNA methylation detection based on methylation-dependent MspJI digestion by characterizing the different types of sequence contexts enriched and analyzing the difference between the common CpG methylation and non-CpG methylation like ^m^CHG and ^m^CHH in plants. We further reason that combining multiple MspJI-like enzymes can allow recognition of a wider set of methylated sites. The DNA modification of hydroxymethylation can also be detected through MspJI-seq based on glycosylation treatment, Thus these modification-dependent restriction endonucleases of MspJI family are thought to be promising in future epigenetic studies.

## Results

### Simulation of enzymatic digestion on *Arabidopsis thaliana* by methylation- dependent restriction endonuclease MspJI

Based on the former study in recognition specificities of the MspJI enzyme [7], we performed *in silico* analysis to simulate MspJI digestion on the *Arabidopsis* genome. In approximate calculation, CNNR loci account for 49% of the total cytosines in *Arabidopsis* genome, assuming they are all methylated. We also applied the *in silico* simulation to the rice genome and more than 50% of the methylated cytosines are also enriched, indicating that our method is feasible for application in other plants.

Depending on the distance between the two closest ^m^CNNR sites and whether the methylated cytosines in these sites are symmetrically or asymmetrically located on the DNA double strands, the enzyme cleavage will occur in different scenarios. Especially, the results of competing cleavage will be decided by the cutting order and the interaction between the two recognition contexts [7], thus it’s difficult to simulate all the possible cleavage products. Here we describe six main cleavage scenarios occuring in ^m^CNNR sites in both CpG and non-CpG contexts (See Methods and Additional file 1. The script of pipeline was uploaded to SourceForge). With this simulation, we calculated the number of potential MspJI recognition sites and found that at least 10.72 M cytosines in fragments ranging from 28 bp to 35 bp can be extracted from the genomic DNA of *Arabidopsis* by MspJI digestion (Two-way cleavage, Additional file 1E, 1F), representing 25.03% of the total 42.86 M cytosines in *Arabidopsis* genome (Table 1). Typically, if MspJI recognizes symmetrically methylated cytosines like ^m^CpG or ^m^CHG sites, the cleavages will result in DNA fragments in length of 32 bp or 31 bp respectively, with methylated sites in the middle [7]. Based on this simulation, we size-selected the major bands approximately ranging from 28-35 bp for library construction and sequencing, in order to profile the representative methylated cytosines in *Arabidopsis* genome. In concern with the mapping problem of these 28-35 bp short sequences, we further simulated the re-alignment of these selected fragments to the reference genome. Averagely more than 99.842% or 90.875% of the fragments can be mapped totally or uniquely back to the genome, respectively, ensuring a high mapping efficiency (Additional file 2).

**Table 1:** The typical sites in two-way cleavage scenarios

**Typical sites**	**Fragments' length(bp)**	**Sites’ number in *Arabidopsis *genome**		**CNNR type**
YNCGNR	32	628107	Total 4194480	Symmetrical CGNR
YCHGR	31	840155		Symmetrical CHGR
CHHG	30	2726218		Symmetrical CHHG
CYHAG	29	452582	Total 1169473	Symmetrical CHHRG
CHYAHG	28	385221		Symmetrical CHHRNG
TDDGCHHA	34	168028		symmetrical YNNGCNNR
TDDGNCHHA	35	163642		Symmetrical YNNGNCNNR
Total cytosines in the sites above		Total cytosines in *Arabidopsis* genome		Generation rate
10727906		42859589		25.03%

The two-way cleavage is caused by the MspJI recognition on the symmetrically methylated sites which are mostly enriched in plant genome. We calculated the typical symmetrical CNNR sites in case of MspJI digestion, and found that these sites contain 10.72 M cytosines which account for 25.03% of the total cytosines in *Arabidopsis* genome. (R = A or G; Y = C or T; H = A or C or T; D = A or G or T).

### Determination of methylated cytosines by MspJI-seq

**Figure 1:**
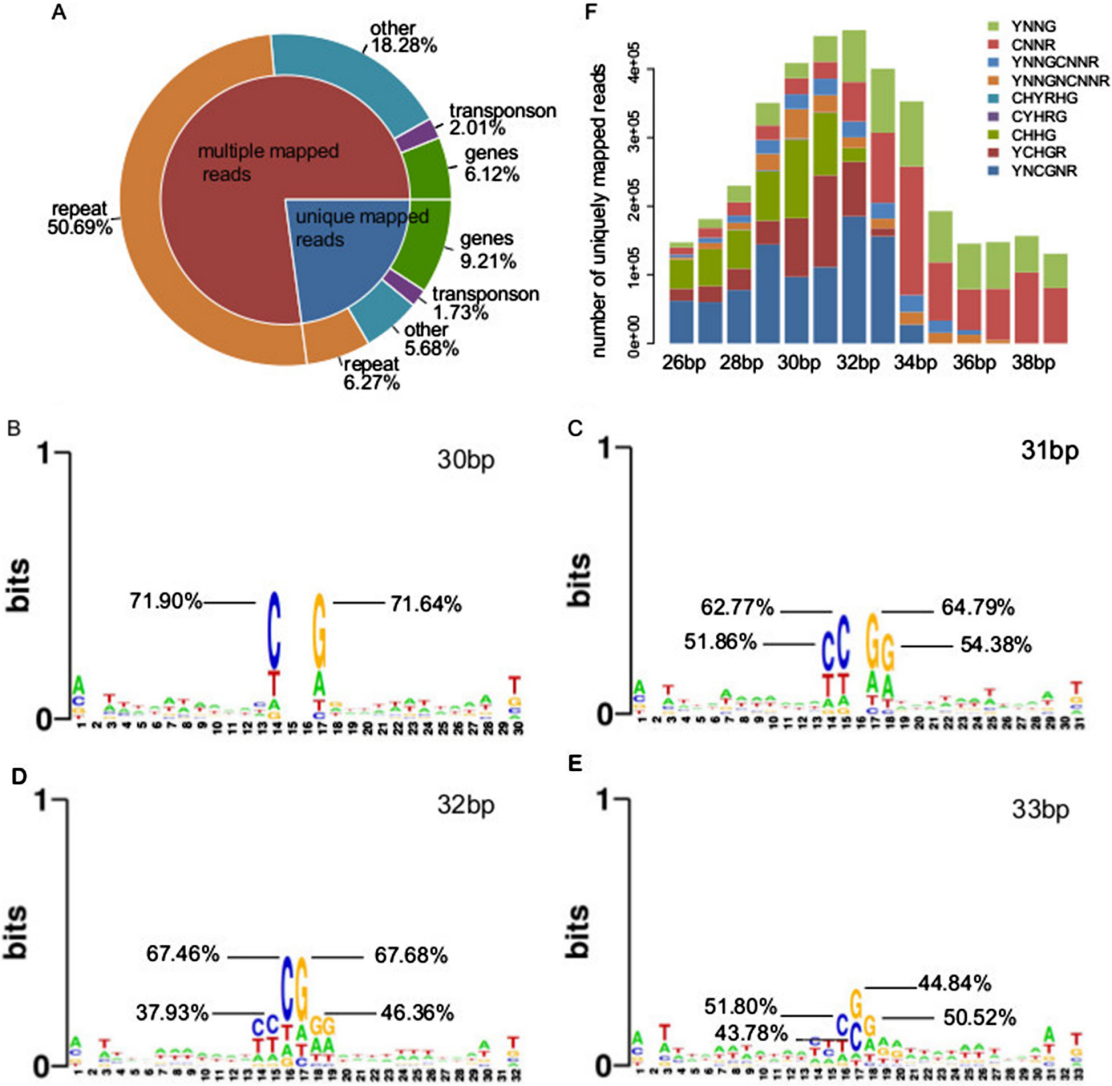
**Features of sequenced reads in MspJI-seq.** (**A**) Distribution of all mapped reads on *Arabidopsis* genome. (**B**-**E**) Logos of insert fragments in length of 30 bp to 33 bp. Sequence characteristics were investigated by randomly extracting 1000 insert fragments in certain length from the uniquely mapped reads. Logos of 30 bp, 31 bp and 32 bp fragments (**B**, **C** and **D**) respectively present specific CHHG, CHG and CpG sites in the middle, and that of the 33 bp fragments (**E**) presents the CpG sites in a wobbling position. (**F**) Site categories in uniquely mapped reads with different length. The fragments in length of 26-33 bp mainly contain the symmetrical ^m^CGNR and ^m^CHGR sites, while fragments in other length mainly contain asymmetrical ^m^CNNR sites. Results in Figure 1 were generated from the data of MspJI-seq replicate 1.

We applied Illumina HiSeq2000 platform to sequence the library comprised of the gel-extracted DNA fragments that were generated from MspJI digestion in *Arabidopsis* genome. As a result of sequencing, 32.1 M clean reads (single-end 50 bp length) were yielded with a mapping rate of 81.65% and a unique mapping rate of 18.69% when mapping to the *Arabidopsis* reference genome (Additional file 3A). By matching with the information of genomic annotation, totally 13.6 M reads (67.43%) of the total 20.2 M reads with multiple mapping positions can be mapped to repetitive regions of *Arabidopsis* genome, which mostly explained the low unique mapping rate and indicated highly methylated repetitive sequences. Among the reads mapped to repetitive sequences, most are located in the satellites of centromeres (highly repetitive sequences) and rRNA genes (moderately repetitive sequences) (Additional file 4), which is in consistence with previous studies suggesting that DNA is heavily methylated in the *Arabidopsis* heterochromatic regions [10,11]. Only a small portion of reads are distributed in genic regions, indicating a relatively lower methylation level of genes in *Arabidopsis* genome (Figure 1A). However, 34575 (99.35%) out of 34802 genes and 4775 (98.70%) out of 4838 pseudogene and transposons were covered by at least one read, providing sufficient coverage information for further functional studies.

The actual length of insert fragments of sequencing reads is ranged from 26 bp to 39 bp after trimming off the sequencing adapters, among which 30 bp to 33 bp are mostly enriched. To determine the methylated cytosine, we notarized CNNR sites that are located in certain distance from the ends of the mapped reads based on our simulation of MspJI recognition sites. As the sequence logos of the fragments indicate, the obtained cytosines either with CpG, CHG or CHH contexts are mostly located in the middle of the insert fragments ranging from 30 bp to 33 bp (Figure 1B-E), suggesting for one ^m^CNNR site inside the fragment. But two ^m^CNNR sites may co-exist inside the fragments (Additional file 1E), or distribute outside the fragments (Additional file 1F), in consideration of which surrounding sequences of the fragments should be included for the determination of ^m^CNNR sites. As a result, we summarize the counts of ^m^CNNR sites in different categories in Figure 1F. Corresponding to the distribution of fragments based on length, the categories of YNCGNR and YCHGR sites are mostly enriched, either in uniquely mapped reads or in all mapped reads in total (Figure 1F, Additional file 5). Thus, the methylated cytosines can be efficiently identified from these sequence contexts, and the number of MspJI fragments can represent the level of methylated cytosines. We then used the uniquely mapped reads in following analysis for the interpretation of methylome of *Arabidopsis*.

### Assessment of MspJI-seq technology

**Figure 2:**
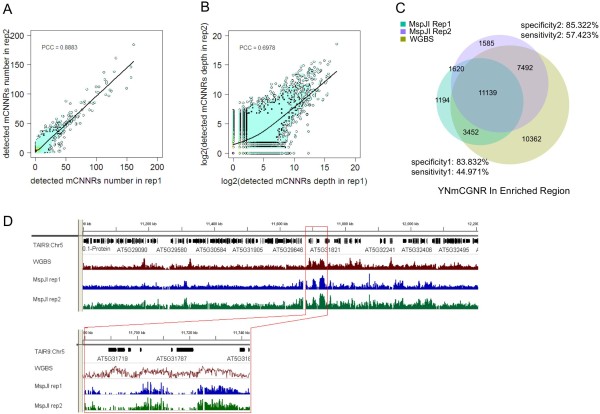
**Assessment of MspJI-seq technology.** (**A**) Pearson correlation analysis of detected ^m^CNNR number on enriched peaks of sequencing reads between two MspJI-seq replicates. (**B**) Pearson correlation analysis of ^m^CNNR depth (methylation level) of enriched peaks between two MspJI-seq replicates. (**C**) Venn diagram indicating the overlap of YNmCGNR sites in enriched peaks detected in two MspJI-seq replicates and WGBS data. The specificity and sensitivity of MspJI method are calculated as the ratio of YNmCGNR sites commonly determined by MspJI-seq and WGBS to the total YNmCGNR sites determined by MspJI-seq or by WGBS, respectively. The two data sets of MspJI-seq replicates were counted separately for the two pairs of specificity and sensitivity. (**D**) The comparison of relative methylation levels on randomly chosen genomic regions (50 bp intervals) between two replicates of MspJI-seq and WGBS data.

To assess the repeatability of MspJI-seq technology, we independently constructed another library for the same *Arabidopsis* sample and conducted high-throughput HiSeq sequencing with the same sequencing strategy. 27.9 M clean reads were yielded with a mapping rate of 70.68% and a unique mapping rate of 9.34% (Additional file 3B). Similarly, majority of the multiply mapped reads can be mapped to repetitive regions of *Arabidopsis* genome. As we quantify the level of methylated cytosines by the number of MspJI fragments, for which sequencing depth or the total data volume could be a factor that distorts the result. To eliminate such influence, we focused our analysis on highly methylated genomic regions. By adapting a model-based anaylsis of ChIP-Seq (MACS) algorithm [12], we identified specific genomic peak regions that were enriched with uniquely mapped MspJI fragments for two replicates of sequencing data. As a result, 11301 peaks were commonly enriched between two replicates. Pearson correlation analysis was then performed between the two sets of data on the numbers of detected ^m^CNNR sites (Figure 2A) and methylation levels of the detected ^m^CNNRs within the enriched peaks (Figure 2B). Pearson Correlation Coefficients (PCC) of 0.8883 and 0.6978 were obtained respectively, indicating an acceptable repeatability of this method.

In order to assess the specificity and sensitivity of the method, we further examined the detected YNCGNR sites among our two replicates of data and a set of WGBS methylome data of *Arabidopsis* downloaded from GEO (accession number GSM399600), requiring that the 32-bp fragments encompassing the symmetrically methylated CGNR sites are unique in the *Arabidopsis* genome [5]. We defined the methylated YN^m^CGNR sites as the ones covered by at least one read in our MspJI data. Similarly, methylated YN^m^CGNR sites in WGBS data were defined as the ones covered by at least one read that supported for methylation in this position. As a result, 0.032M WGBS-detected YNmCGNRs within the 11301 peaks were picked out and used as a reference to validate the MspJI-seq data. On average, 84.58% of the YNmCGNRs in methylation map of MspJI-seq were identified to be methylated in WGBS data, while 51.20% of the YNmCGNRs in WGBS data were detected by MspJI-seq, respectively (Figure 2C). These results indicated satisfactory specificity but less acceptable sensitivity of the MspJI-seq method in comparison with WGBS technology, suggesting more sequencing data might be required to reach a better coverage of methylated cytosines. Additionally, the methylated CNNR sites in WGBS data were determined by the binominal distribution test originally applied by Lister et al. [13] for examination of relative methylation levels, which were defined as the ratios of ^m^CNNR number to CNNR number within genomic regions (Figure 2D, additional file 6). A general concordance of methylation patterns was observed between MspJI-seq and WGBS technologies, therefore indicating the MspJI-seq method is feasible to be applied in methylation profiling of *Arabidopsis* genome.

### Characteristics of *Arabidopsis* methylome

**Figure 3:**
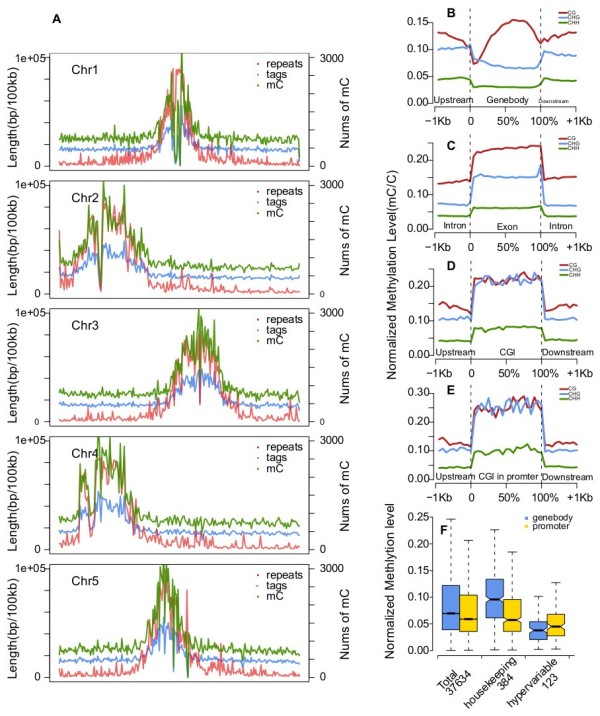
**Characteristics of *Arabidopsis *methylome generated by the combined MspJI-seq data.** (**A**) The density of 5 mCs and repetitive sequences throughout five chromosomes of *Arabidopsis* genome. The density of 5 mCs and MspJI-seq tags were calculated as their numbers in 100 kb intervals, and the density of repeats were calculated as repetitive sequence’s length in 100 kb intervals. (**B**-**E**) The relative methylation levels of genic regions and high-CpG regions (indicated by CGNR\CHGR\CHHR contexts). Each genic or high-CpG region were equally divided into 20 intervals and their 1 kb surrounding sequences were divided into 100 bp intervals. The figures display average methylation levels on intervals of gene bodies (**B**), exons and introns (**C**), CGIs (**D**) and the CGIs overlapping with gene promoters (**E**). (**F**) The normalized methylation levels of housekeeping genes and hypervariable genes. The calculation of relative methylation levels of gene sequences is just as that of intervals.

We then combined the two sets of MspJI-seq data in order to characterize the methylation profile of *Arabidopsis* genome based on uniquely mapped ^m^CNNR sites determined in the combined MspJI-seq data. In chromosome scale, we found a high enrichment of 5 mCs in repeat-rich pericentromeric regions (Figure 3A). In addition, the density of detected 5 mCs was found to correlate with the density of repetitive sequences, which possessed clearly higher methylation levels (^m^CNNR reads density) than genic regions, as suggested by Pablo D et al. [14,15]. Furthermore, we investigated the DNA methylation levels across gene regions. We found that the CpG methylation is depleted at the transcription start site (TSS) and the transcription terminal site (TTS), but maintained at high levels in gene bodies. In contrast, CHG and CHH methylation remain almost unchanged at a low level in gene bodies (Figure 3B). Further examination indicated, within gene bodies, generally higher methylation level were displayed in exons than in introns in all three contexts (Figure 3C), which was in agreement with a previous study that demonstrated such methylation preference on CpG sites by shotgun genomic bisulfate sequencing [16].

We further examined the methylation status in relation with sequence context and found that regions with higher concentrations of CpG dinucleotides are more heavily methylated either at CpG and non-CpG sites (Figure 3D, 3E), which is in agreement with the former discovery [8]. This is interestingly different from mammalian genome, in which the regions with high CpG concentration termed as CpG islands (CGI) are usually hypomethylated and associated with the majority of gene promoters, which makes the CGI methylation affect on transcription initiation [17,18]. However, the extensive DNA methylation on high-CpG regions and its poor intersection with promoters (41.2%) in *Arabidopsis* might indicate much weaker function of CGI methylation in regulation of transcription in comparison with that of mammals.

Next we tested the methylation level of specific genes that were reported to be hypermathylated (housekeeping genes) and hypomethylated (hypervariable genes) in previous study [19]. We normalized the methylation levels of all genes and their promoters in our profile, and found a remarkable disparity that housekeeping genes are hypermathylated and hypervariable genes are hypomethylated on gene bodies, despite the methylation levels of their promoters are both similar to the average level (Figure 3F).

In summary, the characterization of either global methylation pattern or methylation level of specific genes of *Arabidopsis* by MspJI-seq is in consistence with previous discoveries [8,9,19], indicating a general feasibility of the new method in detecting genome-scale methylation profiles.

## Discussion

In present study, we applied MspJI-seq for genome-wide profiling of 5 mCs. This method relies on the MspJI enzyme that can specifically recognize single ^m^CNNR sites *in vivo*. Naturally, it enables a precise detection of 5-mC and quantification of methylation level for a specific CNNR site, providing better resolution in comparison with affinity-based methods, such as methylated DNA immunoprecipitation sequencing (MeDIP-sequencing) and methyl-binding protein sequencing (MBD-seq).

To start with, we optimized the protocol of MspJI digestion on plant genome and recovered a notarized length range (28-35 bp) of fragments based on our simulation of MspJI digestion on *Arabidopsis* genome. By sequencing the recovered fragments, methylcytosines in CNNR context can thus be identified and quantified from the sequencing reads. Data from two independent MspJI-seq experiments were analyzed to estimate the repeatability of the method as well as its specificity and sensitivity in comparison with WGBS data. As a result, we obtained acceptable repeatability and specificity of MspJI-seq in detecting methylated CNNR sites. However, the average technical sensitivity is a bit low (51.20%). Low-frequency methylated CNNR sites in a cell population might be neglected due to insufficiency of sequencing depth, wastage of digested fragments in the process of size-selecting and purification or even the competing cleavage on ^m^CNNRs that are adjacently existed in genomic regions. Nevertheless, MspJI-seq can still be used to identify most of the symmetrical ^m^CNNRs, which are typically enriched in plant genomes.

By defining the methylation levels of genomic regions as the ^m^CNNR density, we portrayed the methylation pattern of different genomic regions. We found that CpG and non-CpG context are differently methylated in gene bodies, and exons display higher methylation levels than introns in both contexts. Our results are consistent with previously published *Arabidopsis* methylome in that high-CpG regions are heavily methylated either at CpG and non-CpG sites in *Arabidopsis* genome [8,9]. Thus it’s feasible to examine the representative methylation profile by using the MspJI-seq method with a reduced data volume. Because of the competing cleavage induced by the nearby ^m^CNNR sites (Additional file 1B, 1D), it will be difficult to attribute methylation levels for high-density 5 mCs within CNNR sites. In that case, it’s especially suitable for determining methylation levels in specific genomic intervals. Furthermore, this MspJI-seq method can be further improved. For instance, a bigger range of digestion products can be recovered in sequencing library construction to ensure the detection of a larger scope of asymmetrically methylated cytosines. In addition, as most of the reads were multiply mapped to repetitive sequences and can’t be used to infer methylation status precisely, removing repetitive sequences prior to sequencing should be considered. One possible approach is to employ subtracting hybridized biotin-labeled repetitive-sequence DNA complex with phenol and chloroform after incubation of hybridized products with avidin, thus producing unique products that are formed after such repetitive sequences have been removed from the DNA [20].

## Conclusions

This work provides an example for combining methylation-depedent enzyme MspJI with high-throughput sequencing in detection of DNA methylation. We emphasize that such digestion-based method is equipped with low cost and high efficiency on representatively determining DNA methylation profiles in all CpG, CHG and CHH contexts, thus the method can be further used in methylome investigating for other species. With proper improvement, the other members of MspJI family can also be introduced in methylation study. Furthermore, it is hoped that MspJI-seq can be used to distinguish hydroxymethylcytosines from methylcytosines, as glycosylation treatment on hydroxymethylated cytosines will hamper the recognition and digestion by MspJI.

## Methods

### Sample preparation

Germinated *Arabidopsis thaliana* seeds (genotype: wild-type, ecotype: Columbia) were grown in 1/4 MS culture medium at 23°C under a 10-hour light/14-hour dark cycle for 13 days. Then seedlings were transplanted to potting soil and grew at 28°C for 45 days. Genomic DNA was extracted from the 45-day *Arabidopsis* leaves using the cetyltrimethylammonium bromide (CTAB) method followed byphenol: chloroform extraction and ethanol precipitation. DNA quality was checked by 1% agarose gel electrophoresis. The genomic DNA was prepared for construction of two replicates of MspJI-seq libraries.

### Library construction and sequencing

1.5 μg genomic DNA was digested at 37°C for 16 h by 12U MspJI enzyme (NEB) in the presence of 0.8 μM double-stranded DNA activator (Invitrogen) in a 30 μl volume. The digestion system was optimized for the *Arabidopsis* genome from the original NEB protocol. By running the digested DNA in a 15% native polyacrylamide gel electrophoresis (PAGE), a narrow-band containing all the visible fragments around 28-35 bp was excised in reference of 10 bp DNA ladder (NEB). DNA was isolated by Crush and Soak Method [21] and purified by ethanol precipitation. Recovered DNA was used to construct sequencing library according to the Illumina Pair-End protocol including procedures of DNA end-repair, ‘A’ base addition, adapters ligation and PCR amplification. Phenol: chloroform extraction and ethanol precipitation were used to purify the products of each process. PCR reaction was fulfilled by JumpStart™ Taq DNA Polymerase (Sigma) for 6 cycles, and its products at length of 148-155 bp were recovered from a 2% agarose gel electrophoresis in reference of 50 bp DNA ladder (NEB), and purified according to QIAquick gel extraction kit (Qiagen). The obtained library was analyzed by Bioanalyzer analysis system (Agilent, Santa Clara, USA) before sequencing with Illumina HiSeq2000.

### Data processing and analysis

We developed a pipeline using Perl for data processing and analysis including simulation of MspJI enzymatic digestion, reads trimming and filtering, reads alignment, sites recognition and ^m^CNNR collection. The scripts can be found online at http://sourceforge.net/projects/mspjiseqpipelin/. Briefly, raw sequencing data was processed by the Illumina base-calling pipeline. Low-quality reads that contained more than 30% ‘N’s or over 10% of the sequence with low quality value (quality value <20) per read were omitted from data analysis. Then the clean reads trimmed off sequencing adapters were aligned to the *Arabidopsis* reference TAIR9 (The *Arabidopsis* Information Resource, http://ftp.arabidopsis.org/home/tair/Genes/TAIR9_genome_release/) using Soap2.20 (http://soap.genomics.org.cn/index.html) [22] with default parameters. In the annotation of mapped reads (Figure 1A, Additional file 4), we randomly picked up one mapping position for multiply mapped read and repeated this sampling for 1000 times, the mean value of the repeats were adopted as final results for this multiply mapped read. The regular matching seeking algorithm in perl was used to identify the CNNR sites within the mapped reads, and the cytosines in notarized CNNR sites obtained by MspJI-seq were determined as the methylated cytosines. The counts of fragments with CNNR sites (the sequencing depths) were defined as the absolute methylation level of the cytosine within the CNNR site and the read density of CNNR sites in a certain genomic region were defined as the relative methylation level of this region. Protein-coding genes, pseudogenes and tansposons were defined from the TAIR9 annotations, genomic repeat regions were mapped by RepeatMasker (version open-3.2.8), and high-CpG regions were detected by Model-based CpG Islands (http://rafalab.jhsph.edu/CGI/) with a posterior probability threshold of 0.99.

Sequence logos of fragments and sites were constructed using weblogo software (http://weblogo.berkeley.edu/), which are graphical representations with the height of symbols within the stack indicating the relative frequency of each nucleic acid at that position. Raw and processed data of two MspJI-seq replicates were uploaded to GEO with the accession number GSE46428.

### Public data used

The WGBS data from aerial tissues of wild-type *Arabidopsis thaliana* (Ecotype Columbia) was downloaded from GEO (accession number GSM399600).

## Competing interests

The authors declare that they have no competing interests.

## Authors’ contributions

FG conceived the project and interpreted data, XH, LX, JS and J-WW performed experiments. HL performed bioinformatics analysis, XH and FG wrote the manuscript with help from SL. All authors read and approved the final manuscript.

## Supplementary Material

Additional file 1 **The MspJI cleavage scenarios.** MspJI cleavage is described as six scenarios determined by the location of the two nearest recognition sites. When the two nearest ^m^CNNR sites lie in the same strand, two cleavages will happen independently if they are at a distance of 12 bp or more, under this circumstance, the length of digestion products which contain only one recognition site will be at a wide range (A), if their distance is lesser than 12 bp, competing cleavage happens to produce nothing but a cutting terminal (B); When the two nearest ^m^CNNR sites are located in the downstream of each others’ cutting direction and in different strands, a fragment with any length but no recognition site will be generated if the two sites are at a distance of 29 bp or more (C), competing cleavage will happen if the distance is from 16 bp to 28 bp (D), two-way cleavage occurs to produce a fragment with two recognition sites at a length of 18 bp to 32 bp, in this case, one ^m^CNNR site is 15 bp or less away from another (E); When the two nearest sites are located in the upstream of each others’ cutting direction and in different strands, the two-way cleavage product also contains two recognition sites and is more than 33 bp in length (F). Click here for file

Additional file 2 **Re-alignment of the selected fragments to the reference *Arabidopsis *genome.** We simulated the alignment of short fragments which were generated by randomly splitting the reference *Arabidopsis* genome into 28-35 bp. On average, 99.842% and 90.875% of the short fragments can be mapped totally or uniquely back to the genome, respectively. Click here for file

Additional file 3 **Mapping results for two MspJI-seq replicates.** The ratios of mapped reads and uniquely mapped reads to the clean reads were defined as the mapping rate and the unique mapping rate respectively. Table A is for replicate 1 and table B is for replicate 2. Click here for file

Additional file 4 **Distribution of sequencing reads in genomic repeat regions.** (A) Distribution of multiply mapped reads in repetitive sequences. (B) Distribution of uniquely mapped reads in repetitive sequences. Results in Additional file 3 were generated from the data of MspJI-seq replicate 1. Click here for file

Additional file 5 **Site categories in all mapped reads with different length.** The fragments in length of 26-33 bp mainly contain the symmetrical ^m^CGNR and ^m^CHGR sites, while fragments in other length mainly contain asymmetrical ^m^CNNR sites. Results were generated from the data of MspJI-seq replicate 1. Click here for file

Additional file 6 **Relative methylation levels on several candidate genes.** It’s a comparison of relative methylation levels (checked by 50 bp intervals) between MspJI-seq replicates and WGBS data. Click here for file
