# Supplementary figures and images for: High-throughput sequencing of methylated cytosine enriched by modification-dependent restriction endonuclease MspJI
Source: BMC Genet. 2013 Jun 18;14:56. doi: 10.1186/1471-2156-14-56 (PMC3718668; doi:10.1186/1471-2156-14-56)

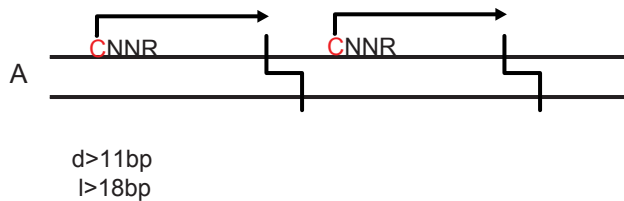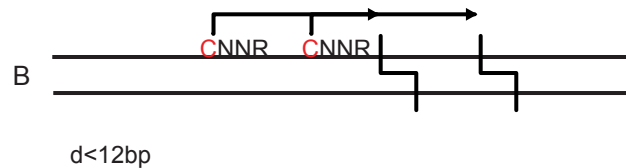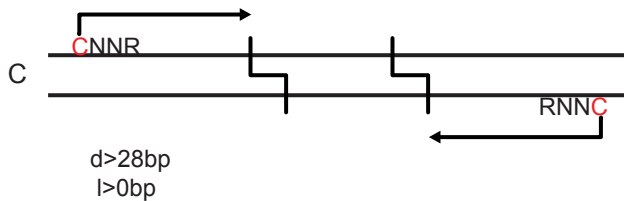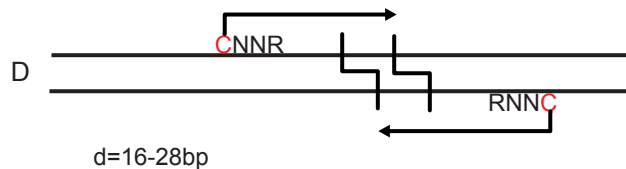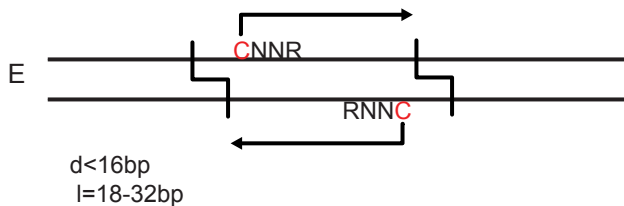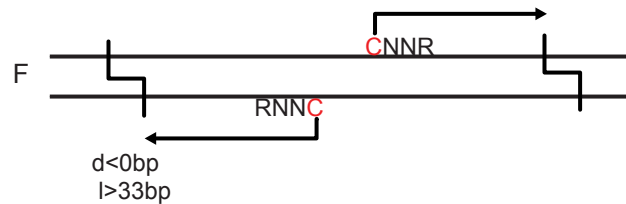

Supplement: Additional file 1 — The MspJI cleavage scenarios. MspJI cleavage is described as six scenarios determined by the location of the two nearest recognition sites. When the two nearest mCNNR sites lie in the same strand, two cleavages will happen independently if they are at a distance of 12 bp or more, under this circumstance, the length of digestion products which contain only one recognition site will be at a wide range (A), if their distance is lesser than 12 bp, competing cleavage happens to produce nothing but a cutting terminal (B); When the two nearest mCNNR sites are located in the downstream of each others’ cutting direction and in different strands, a fragment with any length but no recognition site will be generated if the two sites are at a distance of 29 bp or more (C), competing cleavage will happen if the distance is from 16 bp to 28 bp (D), two-way cleavage occurs to produce a fragment with two recognition sites at a length of 18 bp to 32 bp, in this case, one mCNNR site is 15 bp or less away from another (E); When the two nearest sites are located in the upstream of each others’ cutting direction and in different strands, the two-way cleavage product also contains two recognition sites and is more than 33 bp in length (F). [file 1471-2156-14-56-S1.pdf]

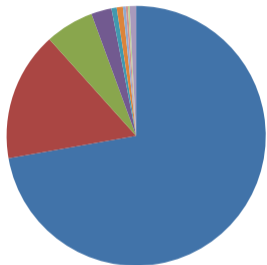

A

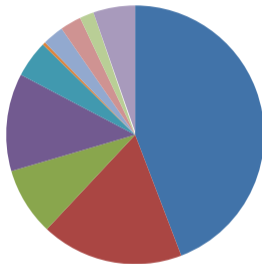

B

- Satellite/centr
- rRNA
- Satellite
- LTR/Gypsy
- DNA/MuDR
- LTR
- RC/Helitron
- LTR/Copia
- DNA/En-Spm
- other

Supplement: Additional file 4 — Distribution of sequencing reads in genomic repeat regions. (A) Distribution of multiply mapped reads in repetitive sequences. (B) Distribution of uniquely mapped reads in repetitive sequences. Results in Additional file 3 were generated from the data of MspJI-seq replicate 1. [file 1471-2156-14-56-S4.pdf]

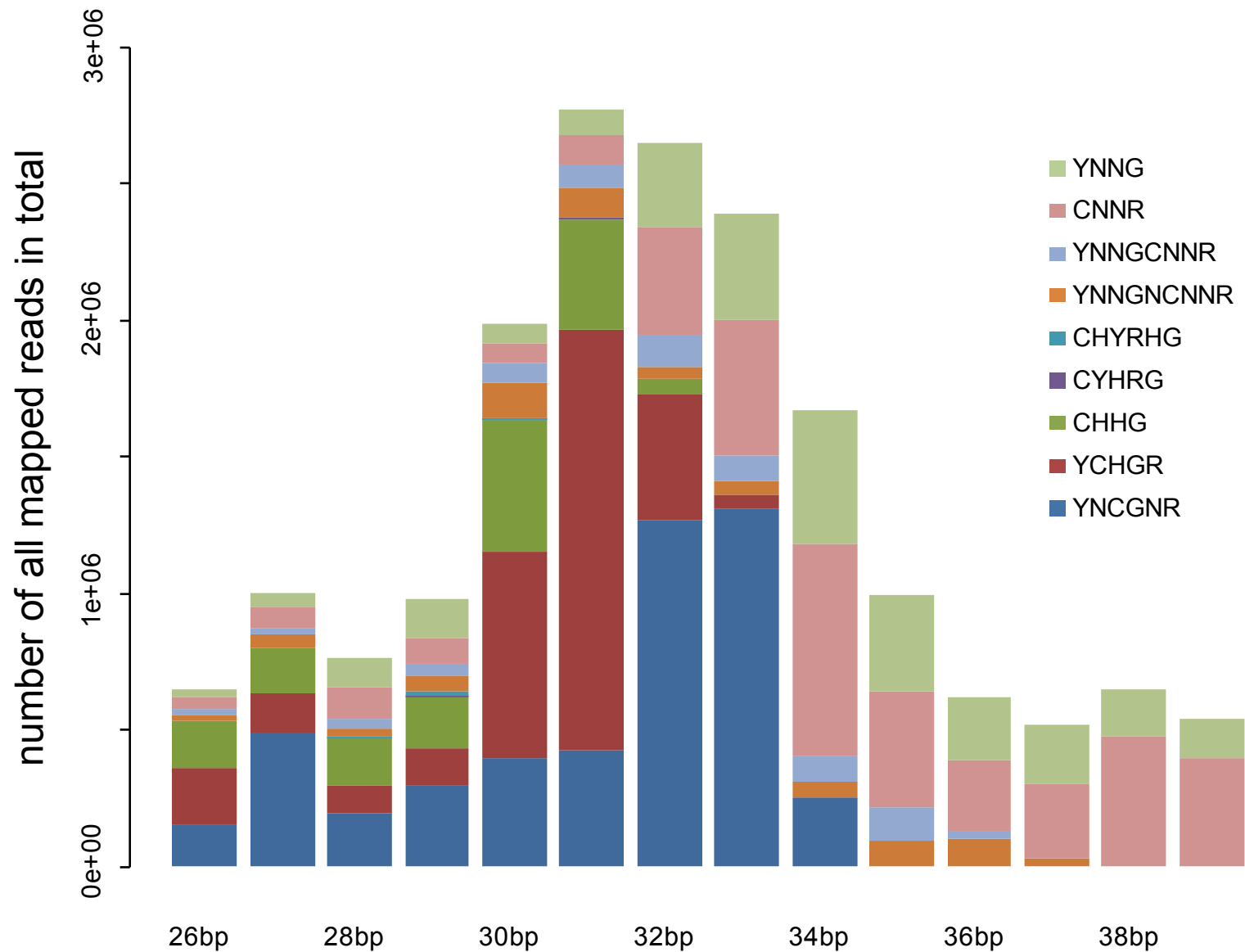

Supplement: Additional file 5 — Site categories in all mapped reads with different length. The fragments in length of 26-33 bp mainly contain the symmetrical mCGNR and mCHGR sites, while fragments in other length mainly contain asymmetrical mCNNR sites. Results were generated from the data of MspJI-seq replicate 1. [file 1471-2156-14-56-S5.pdf]

A

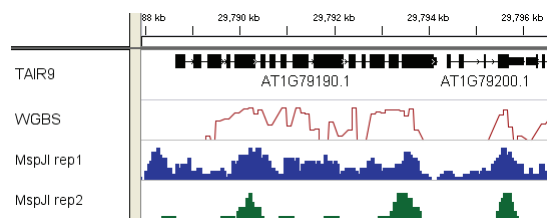

B

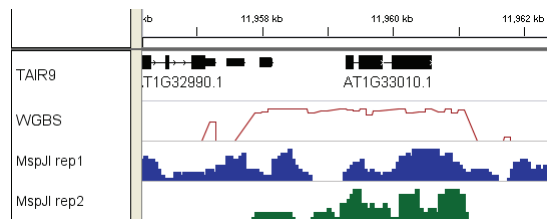

C

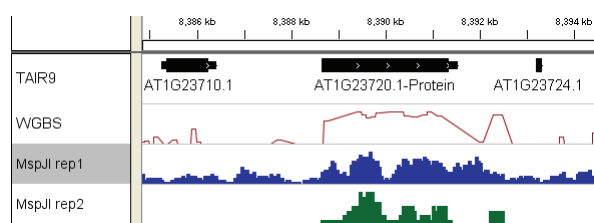

D

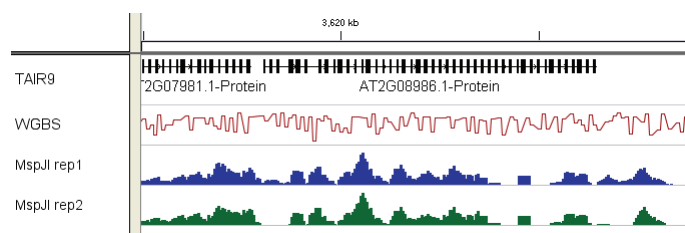

E

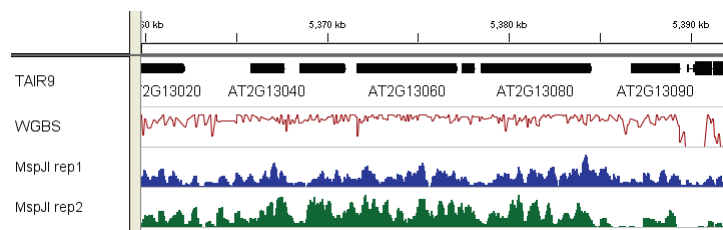

F

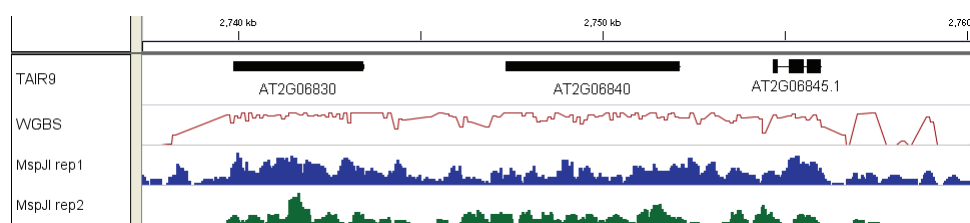

G

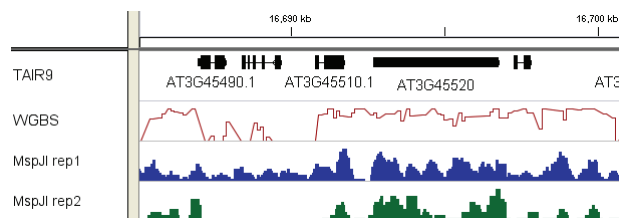

H

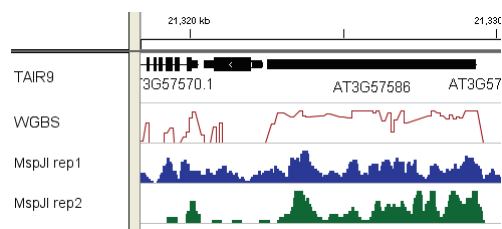

I

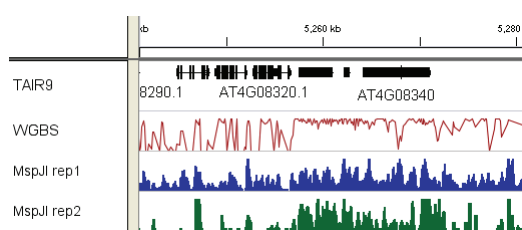

J

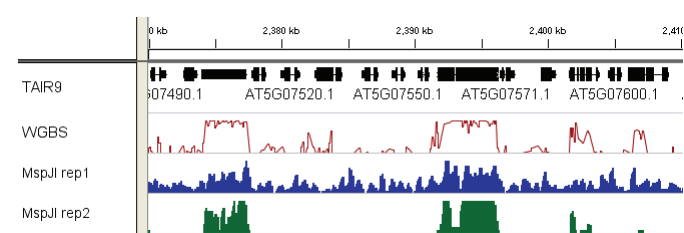

K

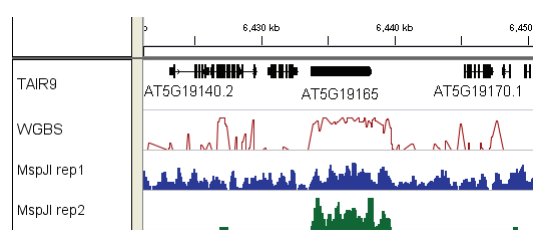

Supplement: Additional file 6 — Relative methylation levels on several candidate genes. It’s a comparison of relative methylation levels (checked by 50 bp intervals) between MspJI-seq replicates and WGBS data. [file 1471-2156-14-56-S6.pdf]
