# Supplementary material for: High-throughput sequencing of methylated cytosine enriched by modification-dependent restriction endonuclease MspJI
Source: BMC Genet. 2013 Jun 18;14:56. doi: 10.1186/1471-2156-14-56 (PMC3718668; doi:10.1186/1471-2156-14-56)
Supplement: Additional file 2 — Re-alignment of the selected fragments to the reference Arabidopsis genome. We simulated the alignment of short fragments which were generated by randomly splitting the reference Arabidopsis genome into 28-35 bp. On average, 99.842% and 90.875% of the short fragments can be mapped totally or uniquely back to the genome, respectively. [file 1471-2156-14-56-S2.pdf]

fragments length	total fragments	mapped fragments	mapping rate	uniquely mapped fragments	unique mapping rate
28bp	119,667,533	119,478,663	99.8422%	108,818,562	90.9341%
29bp	119,667,554	119,478,998	99.8424%	107,972,698	90.2272%
30bp	119,667,547	119,478,887	99.8423%	108,272,453	90.4777%
31bp	119,667,540	119,478,773	99.8423%	108,552,848	90.7120%
32bp	119,667,533	119,478,663	99.8422%	108,818,562	90.9341%
33bp	119,667,526	119,478,543	99.8421%	109,071,669	91.1456%
34bp	119,667,519	119,478,433	99.8420%	109,310,307	91.3450%
35bp	119,667,512	119,478,320	99.8419%	109,537,830	91.5351%
