# Supplementary material for: High-throughput sequencing of methylated cytosine enriched by modification-dependent restriction endonuclease MspJI
Source: BMC Genet. 2013 Jun 18;14:56. doi: 10.1186/1471-2156-14-56 (PMC3718668; doi:10.1186/1471-2156-14-56)
Supplement: Additional file 3 — Mapping results for two MspJI-seq replicates. The ratios of mapped reads and uniquely mapped reads to the clean reads were defined as the mapping rate and the unique mapping rate respectively. Table A is for replicate 1 and table B is for replicate 2. [file 1471-2156-14-56-S3.pdf]

Table A

Clean Reads		
32107319(100%)		
Mapped reads		Unmapped reads
26215180(81.65%)		5892139
Uniquely mapped reads	Multiply mapped reads	
6002218(18.69%)	20212899	
Uniquely mapped reads in repeat	Multiply mapped reads in repeat	
1955328	13629943	

Table B

Clean Reads		
27914133(100%)		
Mapped reads		Unmapped reads
19730492(70.68%)		8183641
Uniquely mapped reads	Multiply mapped reads	
2607915(9.34%)	17122577	
Uniquely mapped reads in repeat	Multiply mapped reads in repeat	
1930035	10655456	
